# Role of c-MET Inhibitors in Overcoming Drug Resistance in Spheroid Models of Primary Human Pancreatic Cancer and Stellate Cells

**DOI:** 10.3390/cancers11050638

**Published:** 2019-05-08

**Authors:** Omidreza Firuzi, Pei Pei Che, Btissame El Hassouni, Mark Buijs, Stefano Coppola, Matthias Löhr, Niccola Funel, Rainer Heuchel, Ilaria Carnevale, Thomas Schmidt, Giulia Mantini, Amir Avan, Luciano Saso, Godefridus J. Peters, Elisa Giovannetti

**Affiliations:** 1 Medicinal and Natural Products Chemistry Research Center, Shiraz University of Medical Sciences, 71348-14336 Shiraz, Iran; firuzio@sums.ac.ir; 2 Department of Medical Oncology, Cancer Center Amsterdam, Amsterdam UMC, VU University Medical Center (VUmc), 1081 HV, Amsterdam, The Netherlands; p.che@vumc.nl (P.P.C.); b.elhassouni@vumc.nl (B.E.H.); m.j.n2.buijs@student.vu.nl (M.B.); ilaria187@gmail.com (I.C.); g.mantini@vumc.nl (G.M.); g.j.peters@vumc.nl (G.J.P.); 3 Physics of Life Processes, Huygens-Kamerlingh Onnes Laboratory, Leiden University, 2333 CA, Leiden, The Netherlands; coppola@physics.leidenuniv.nl (S.C.); Schmidt@physics.leidenuniv.nl (T.S.); 4 Division of Surgery, CLINTEC, Karolinska Institutet, SE-171, Stockholm, Sweden; matthias.lohr@ki.se (M.L.); rainer.heuchel@ki.se (R.H.); 5 Cancer Pharmacology Lab, AIRC Start Up Unit, University of Pisa, 56124 Pisa, Italy; niccola.funel@gmail.com; 6 Metabolic syndrome Research center, Mashhad University of Medical Sciences, 91778-99191 Mashhad, Iran; AvanA@mums.ac.ir; 7 Department of Physiology and Pharmacology "Vittorio Erspamer", Sapienza University, 00185, Rome, Italy; luciano.saso@uniroma1.it; 8 Fondazione Pisana per la Scienza, 56017, Pisa, Italy

**Keywords:** pancreatic cancer, three-dimensional culture, drug resistance, cancer-associated fibroblasts, primary cultures

## Abstract

Pancreatic stellate cells (PSCs) are a key component of tumor microenvironment in pancreatic ductal adenocarcinoma (PDAC) and contribute to drug resistance. c-MET receptor tyrosine kinase activation plays an important role in tumorigenesis in different cancers including PDAC. In this study, effects of PSC conditioned medium (PCM) on c-MET phosphorylation (by immunocytochemistry enzyme-linked immunosorbent assay (ELISA)) and drug response (by sulforhodamine B assay) were investigated in five primary PDAC cells. In novel 3D-spheroid co-cultures of cyan fluorescence protein (CFP)-firefly luciferase (Fluc)-expressing primary human PDAC cells and green fluorescence protein (GFP)-expressing immortalized PSCs, PDAC cell growth and chemosensitivity were examined by luciferase assay, while spheroids’ architecture was evaluated by confocal microscopy. The highest phospho-c-MET expression was detected in PDAC5 and its subclone sorted for “stage specific embryonic antigen-4” (PDAC5 (SSEA4)). PCM of cells pre-incubated with PDAC conditioned medium, containing increased hepatocyte growth factor (HGF) levels, made PDAC cells significantly more resistant to gemcitabine, but not to c-MET inhibitors. Hetero-spheroids containing both PSCs and PDAC5 (SSEA4) cells were more resistant to gemcitabine compared to PDAC5 (SSEA4) homo-spheroids. However, c-MET inhibitors (tivantinib, PHA-665752 and crizotinib) were equally effective in both spheroid models. Experiments with primary human PSCs confirmed the main findings. In conclusion, we developed spheroid models to evaluate PSC–PDAC reciprocal interaction, unraveling c-MET inhibition as an important therapeutic option against drug resistant PDAC.

## 1. Introduction

With a five-year survival rate of only 8%, pancreatic ductal adenocarcinoma (PDAC) has the worst prognosis among major malignancies [1,2]. One of the main causes for this dismal prognosis, which has only slightly changed in the past 50 years, is the inherent resistance of PDAC to available therapies. This prompts the re-evaluation of peculiar aspects of PDAC that may contribute to its chemoresistance and lead to almost invariable therapeutic failure [2,3,4].

The specific microenvironment of PDAC, characterized by an abundance of stroma, is one of the suspects that may greatly influence the response to treatment and even cause early metastasis [5,6,7]. The stroma, a collective term for extracellular matrix (ECM) and non-tumor cells, constitutes a major part of the tumor mass in PDAC and recent evidence has shown that in addition to serving as a physical barrier to drug delivery, it also promotes tumor growth and metastasis [8,9,10]. Although there has been some debate initially on whether stroma restrains or supports PDAC cells [11], recent evidence clearly demonstrates the supportive role of stromal cells on PDAC growth and chemoresistance and suggests the usefulness of developing new stroma-targeted therapies [12,13,14].

The PDAC microenvironment contains several cell types, among which the pancreatic stellate cells (PSCs) seem to have very strong influence on the biological behavior of the tumor cells and represent the main source of cancer-associated fibroblasts (CAFs) [15,16]. PSCs are myofibroblast-like cells normally found in the exocrine areas of the pancreas, which migrate and relocate to the tumor mass as a consequence of the inflammation produced by cancer cells [16]. These cells are mainly responsible for secretion of ECM components causing the density of stroma [17]. Recent evidence has shown that PSCs have a crucial impact on invasion of PDAC cells [18], create an ECM barrier impeding the delivery of chemotherapeutics to cancer cells [9], scavenge and metabolize cytotoxic drugs and prevent their delivery to PDAC cells [19] and notably promote the growth and survival of PDAC cells via paracrine secretion of growth factors and other signaling molecules [20,21].

In this context, disease models that encompass the interactions between PDAC cells and PSCs as main players of the tumor-promoting microenvironment may have considerable advantages over conventional, often reductionist methodologies. These models can indeed provide higher biological relevance for testing the efficacy of novel therapies [15].

Furthermore, several reports have illustrated that three-dimensional (3D) culture systems are generally more chemo-/radio-resistant compared to two-dimensional (2D) monolayer cell cultures, and represent more physiologically relevant PDAC models for cancer drug discovery [22]. Indeed, the utter therapeutic failure of almost all pharmacological interventions in PDAC patients may in part be due to the lack of preclinical models that recapitulate the complex biology of PDAC including the 3D cellular organization [23,24].

A substantial body of evidence has shown that c-MET receptor tyrosine kinase plays a crucial role in cancer cell survival, growth and metastasis and it is overexpressed or mutated in certain types of cancer [25,26]. An increased *MET* mRNA expression has been correlated with an unfavorable outcome in PDAC patients (www.R2.amc.nl accessed on 10-04-2019, Supplemental Figure S1). Therefore, c-MET represents an attractive candidate target for discovery of anticancer therapeutics in PDAC and other malignancies [7,27,28,29]. Recent evidence that shows the paracrine source of hepatocyte growth factor (HGF) in the PDAC microenvironment to be mainly secreted by PSCs, further supports the premise that c-MET targeting could be effective not only by directly attacking cancer cells, but also by breaking the dangerous liaison between PSCs and PDACs [21,30,31]. 

In this report, in addition to the use of primary PDAC cells, we took advantage of two important breakthroughs in the field of pancreatic cancer research, i.e., the concomitant use of PSCs grown together with cancer cells as well as the application of 3D spheroid culture systems. The PSC/PDAC hetero-spheroids developed in this study represent an important tool for screening of cancer- and stroma-targeted drugs and the results obtained by this preclinical model showed that targeting c-MET receptor may prove efficacious as a valuable therapeutic strategy in selected cases of PDAC.

## 2. Results

### 2.1. c-MET and Phospho-c-MET Expression in PDAC Cells

**Figure 1:**
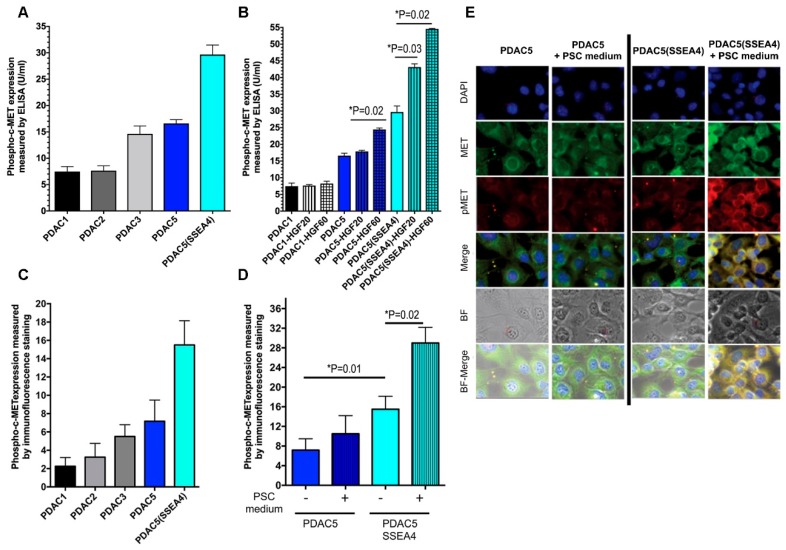
Expression of phospho-c-MET in human primary pancreatic ductal adenocarcinoma (PDAC) cells. Human primary PDAC cells isolated from PDAC patients (PDAC1, 2, 3, 5 and PDAC5 (SSEA4)) were grown in six-well plates for 24 h. Total proteins were extracted from PDAC cells and subjected to analysis by ELISA specific for phosphorylated tyrosine residues 1230, 1234, and 1235 (**A**). PDAC1, PDAC5 and PDAC5 (SSEA4) cells were seeded in six-well plates. After 24 h of incubation, HGF was added at 20 and 60 pg/mL and the cells were further incubated for 24 h. Total protein was extracted and phospho-c-MET levels were measured by the same ELISA kit as described above (**B**). PDAC cells were seeded in 8-chamber slides and after being incubated with PSC conditioned medium for 24 h, were fixed and stained with specific monoclonal rabbit anti-human c-MET and anti-phospho-Y1003-c-MET antibodies (1:200 dilution; Santa Cruz Biotechnology, Dallas, TX, USA). Quantification of immunofluorescence stainings of baseline phospho-c-MET expression (**C**) and after being stimulated with PSC conditioned medium (**D**) are shown using the imaging program AxioVision (Carl Zeiss Microscopy, Jena, Germany). Representative examples (original magnification, 40×) are shown that demonstrate the expression of c-MET and phospho-c-MET in PDAC5 and PDAC5 (SSEA4) cells in the absence or presence of PSC conditioned medium (**E**). DAPI was used to visualize nuclear DNA. Abbreviations: PDAC, pancreatic ductal adenocarcinoma; ELISA, enzyme-linked immunosorbent assay; SSEA4, stage specific embryonic antigen-4; HGF, hepatocyte growth factor; PSC, pancreatic stellate cell; DAPI, 4′,6-Diamidino-2-phenylindole; BF, bright field.

To assess c-MET and phospho-c-MET expression in primary PDAC cells (PDAC1, PDAC2, PDAC3 and PDAC5), we used specific enzyme-linked immunosorbent assay (ELISA), while RNA-sequencing data were used to evaluate c-MET mRNA expression (reported in Supplemental Figure S2). As shown in Figure 1A, ELISA assays specific for phospho-tyrosine residues 1230, 1234 and 1235 showed that PDAC5 and PDAC5 cells sorted for “stage specific embryonic antigen-4” (PDAC5 (SSEA4)), which is a human ductal stem cell marker as detailed in the Supplemental Methods, had the highest baseline phospho-c-MET intensity. Standard curves of measured phospho-c-MET and c-MET as well baseline levels of c-MET protein in PDAC cells are shown in Supplemental Figure S3.

Moreover, phospho-c-MET was measured in PDAC1, PDAC5 and PDAC5 (SSEA4) cells also after incubation with HGF (Figure 1B). A significant increase was observed in PDAC5 cells after incubation with 20 pg/mL and in PDAC5 (SSEA4) after incubation with 20 and 60 pg/mL of HGF. PDAC1 did not show any significant response to HGF stimulation (Figure 1B).

c-MET and phospho-c-MET expression were also further analyzed in primary PDAC cells by immunofluorescence staining and the images were quantified using the specific AxioVision imaging software (Carl Zeiss Microscopy, Jena, Germany), by drawing vectors to quantify the signal of each color channel (red and green). The length of a vector was equal to 6 µm, and it analyzed 40 different points/cell, (Figure 1C). We observed that PDAC5 cells had the highest baseline phospho-c-MET intensity. Remarkably, when PDAC5 and PDAC5 (SSEA4) cells were incubated with PSC conditioned medium (PCM), two-fold higher expression of phospho-c-MET was observed in PDAC5 (SSEA4) cells (Figure 1D,E).

### 2.2. Quantification of HGF in PSC Conditioned Medium

**Figure 2:**
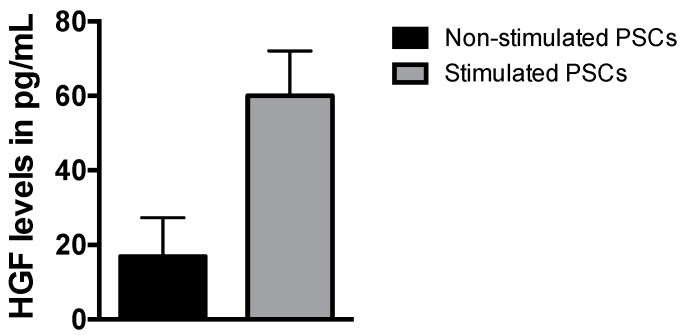
Measurement of HGF levels in medium of (non-)stimulated PSCs. HGF levels (in pg/mL) were measured by ELISA in PSC media, that were previously stimulated with(out) the conditioned medium of primary PDAC5 cells sorted SSEA4 (PDAC5 (SSEA4)) for 3 days. The values shown in the bar chart represent the mean ± standard error of mean (S.E.M.) of at least three experiments. Abbreviations: HGF, hepatocyte growth factor; PSCs, pancreatic stellate cells; ELISA, enzyme-linked immunosorbent assay; PDAC, pancreatic ductal adenocarcinoma; SSEA4, stage specific embryonic antigen-4.

Since hepatocyte growth factor (HGF) is the natural ligand for c-MET receptor and also an important growth factor that has been previously reported to induce tumor growth and drug resistance in cancer cells, we performed HGF measurements in PSC media. The levels of HGF secreted in the medium of stimulated and non-stimulated (base-line) PSCs after 3 days were quantified by a specific ELISA assay. PSCs were stimulated by incubation with PDAC5 (SSEA4) cell conditioned medium for 3 days. These quantifications showed that secreted HGF levels were 3.5 times higher in stimulated PSCs compared to non-stimulated cells (60.0 ± 12.0 versus 16.9 ± 10.4 pg/mL; means ± standard error of mean (S.E.M.) of triplicate measurements) (Figure 2). Of note, these HGF levels were similar to the levels we detected in our exploratory analyses in 10 PDAC patients, showing a trend toward significantly higher values (p = 0.13) in patients who progressed compared to patients with stable disease (Supplemental Figure S9).

### 2.3. Effect of Stimulated PSC Conditioned Medium on Drug Response of PDAC Cells in Monolayer Culture

**Figure 3:**
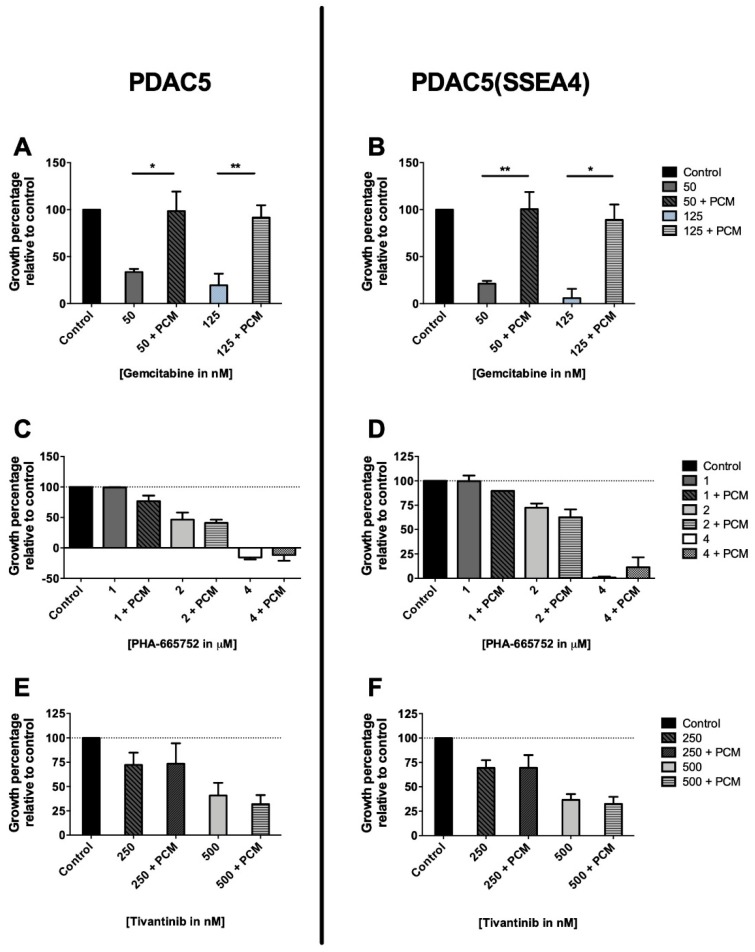
Effect of stimulated PSC conditioned medium on drug response in primary PDAC cells grown in monolayer culture assessed by sulforhodamine B (SRB) assay. PDAC5 and PDAC5 cells sorted for SSEA4 (PDAC5 (SSEA4)) were seeded in 96-well flat-bottom microplates and after incubation at 37 °C overnight, were treated with different drug concentrations in triplicate. Stimulated PSC conditioned medium (PCM) was added to the proper wells in a 1:10 dilution right before drug treatment. The SRB assay was performed after 72 h. The presence of PCM induced significant resistance to gemcitabine (**A**,**B**), while it did not induce any change in drug response against c-MET inhibitors, PHA-665752 (**C**,**D**), and tivantinib (**E**,**F**), in PDAC5 and PDAC5 (SSEA4) cells. The values shown in bar charts represent the mean ± standard error of mean (S.E.M) of at least three experiments (* and **: the difference between cells treated with gemcitabine alone and PCM plus gemcitabine was significantly different at *p* < 0.05 and *p* < 0.01, respectively). Abbreviations: PSC, pancreatic stellate cell; PDAC, pancreatic ductal adenocarcinoma; SRB, sulforhodamine B; SSEA4, stage specific embryonic antigen-4; PCM, pancreatic stellate cell-conditioned medium.

The effect of stimulated PCM on drug response of PDAC cells was examined according to the workflow outlined in Supplemental Figure S4. In the presence of PCM, both PDAC5 and PDAC5 (SSEA4) cells were significantly resistant to the cytotoxic effect of gemcitabine at 50 and 125 nM (Figure 3A,B). Interestingly, this drug resistance was not observed against the c-MET inhibitors tivantinib (at 250 and 500 nM; Figure 3C,D) and PHA-665752 (at 1, 2 and 4 µM, Figure 3E,F).

### 2.4. Formation of Homo- and Hetero-Spheroids in 96-Well Cell Repellant Plates

**Figure 4:**
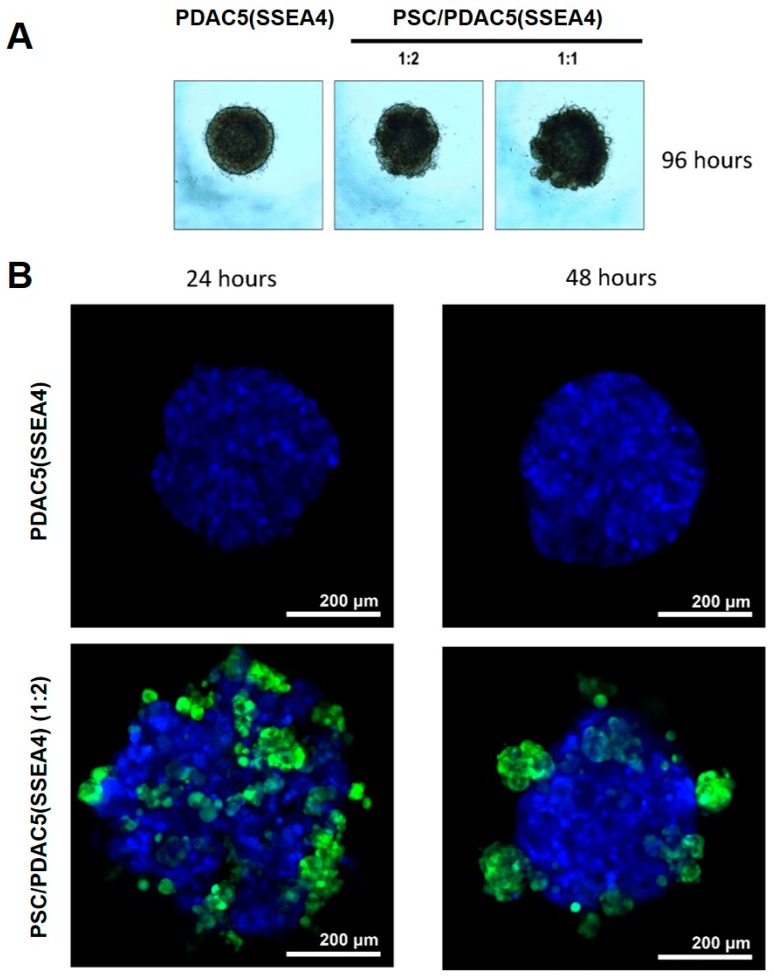
Formation of homo- and hetero-spheroids in cell repellant 96-well plates evaluated by bright field and confocal microscopy. Primary PDAC5 (SSEA4) cells alone or mixed with immortalized PSC at different ratios were grown in 96-well cell repellant plates to form homo- and hetero-spheroids, respectively. All cells were first adapted to serum-free conditions for at least 2 weeks before the start of the experiments. Single spheroids were formed in each well. Representative images were taken on day 4 (**A**). Further images of PDAC5 (SSEA4) and PSC/PDAC5 (SSEA4) cells in homo- and hetero-spheroids were recorded by confocal microscopy, using serum-free adapted CFP-labeled PDAC5 (SSEA4) cells and immortalized GFP-labeled PSCs growing as described in previous figures. The confocal images are maximum intensity projections of four consecutive single focal planes (1 μm apart), selected from the three-dimensional z-stacks (100 μm with 1 μm steps). The corresponding three-dimensional reconstructions are shown in Supplemental Figure S6. Representative images of homo-spheroids of PDAC5 (SSEA4) cells (**B**), and of hetero-spheroids of PSCs and PDAC5 (SSEA4) cells (at a ratio of 1:2, respectively) (**C**), were taken at 24 and 48 h after plating the cells. Of note, after 24h the spheroid has already started to form (Scale bar: 200 μm). Abbreviations: PDAC, pancreatic ductal adenocarcinoma; SSEA4, stage specific embryonic antigen-4; PSC, pancreatic stellate cell; CFP, cyan fluorescent protein; GFP, green fluorescent protein.

For the formation of homo- and hetero-spheroids, either PDAC5 (SSEA4) alone or the mixture of PDAC5 (SSEA4) and PSCs, respectively, were grown in 96-well cell repellent plates at different ratios (PSC to PDAC5 (SSEA4) ratio: 1:8 to 1:1). All cells were first adapted to serum-free conditions over the course of at least two weeks before the experiments. Single spheroids were formed in each well (Figure 4A and Supplemental Figure S5).

### 2.5. Confocal Imaging of 3D Cultures

As a result of the stable cyan fluorescence protein (CFP) and green fluorescence protein (GFP) expression in PDAC5 (SSEA4) and PSCs, respectively, we were able to visualize the structure of the homo- and hetero-spheroids with a confocal microscope (Figure 4B, C). After 24 h, the formation of spheroids was observed with the PSCs (green) forming small clusters between the PDAC5 (SSEA4) cells (blue). Interestingly, 48 h after seeding, PSCs seemed to migrate towards the surface of PDAC5 (SSEA4) spheroids and appeared to form clusters, indicating that a structural reorganization of the spheroid took place within 24–48 h after seeding. Another potential explanation would be the death of cells located in the spheroid interior, though we did not detect debris, which is typically observed after cell death, outside the spheroids. 

### 2.6. Growth and Drug Response of PDAC Cells in the Presence of Immortalized PSCs in 3D Cultures Determined by Luciferase Assay

**Figure 5:**
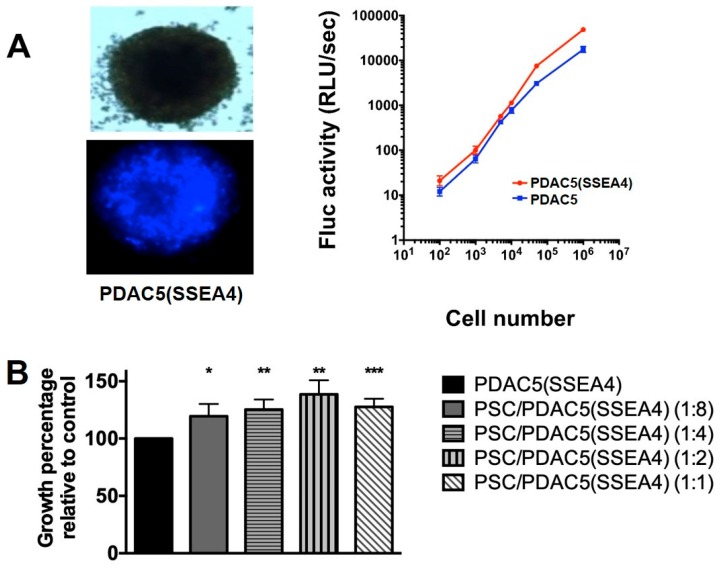
Evaluation of homo- and hetero-spheroids by luciferase assay. Representative images of a homo-spheroid of primary PDAC5 cells sorted for SSEA4 (PDAC5 (SSEA4)) (**A**, left upper image, bright field; left lower image, fluorescence microscopy for CFP. Analysis of luminescence showed that the increase in the BLI signal of Fluc correlates directly with the increasing number of cells (**A**, right panel); y-axis: relative light units per second (Rlu/s). This experiment corresponds to a single time point (i.e., 24 h after seeding), with different cell seeding densities. Effect of PSCs on proliferation of PDAC5 (SSEA4) cells in 3D culture was determined by luciferase assay and compared to homo-spheroids containing the same number of cancer cells (**B**). PSCs significantly increased PDAC5 (SSEA4) cell growth in PSC/PDAC5 (SSEA4) hetero-spheroids. Data show the mean ± standard error of mean (S.E.M.) of at least four experiments (*, ** and ***: the difference between the hetero-spheroid and homo-spheroid consisting of the same number of cancer cells was significantly different at *p* < 0.05, *p* < 0.01 and *p* < 0.005, respectively). Abbreviations: PDAC, pancreatic ductal adenocarcinoma; SSEA4, stage specific embryonic antigen-4; CFP, cyan fluorescent protein; BLI, bioluminescence imaging; Fluc, firefly luciferase; PSCs, pancreatic stellate cells.

The influence of PSCs on the growth of cancer cells as well as the effect of different drugs against homo-spheroids (PDAC5 (SSEA4)) and hetero-spheroids (PSC/PDAC5 (SSEA4)) were examined in 3D cultures in serum free conditions by determination of Firefly luciferase (Fluc) activity, according to the workflow outlined in the Supplemental Figure S7. Fluc was indeed stably expressed in PDAC cells and enabled the quantification of viable tumor cells in spheroids. As reported in the Figure 5A, the firefly luciferase (Fluc) bioluminescence imaging (BLI) signal correlated proportionately with the cell number for both PDAC5 and PDAC5 (SSEA4).

Hetero-spheroids were formed in ratios of 1:8, 1:4, 1:2 and 1:1 (PSC to PDAC ratios, respectively). After 7 days of incubation, we determined the growth percentage of PDAC5 (SSEA4) cells in hetero-spheroids relative to PDAC5 (SSEA4) homo-spheroids, which did not contain PSCs. The number of viable PDAC5 (SSEA4) cells in hetero-spheroids was significantly increased compared to the homo-spheroids containing the same number of cancer cells (Figure 5B).

**Figure 6:**
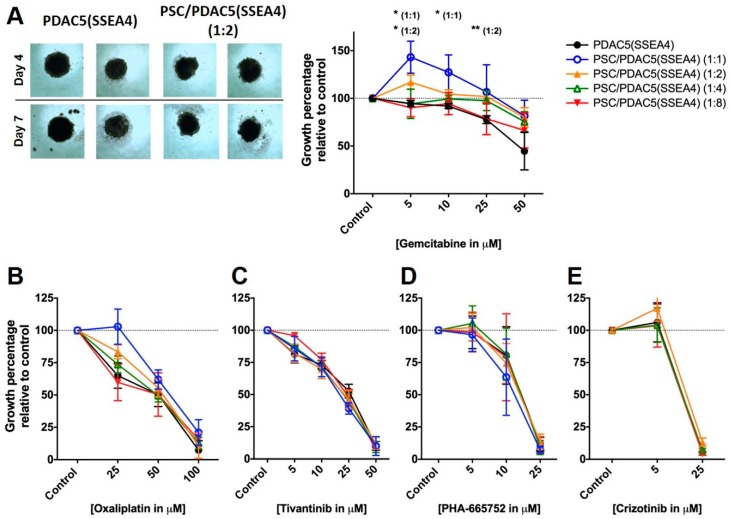
Drug response in homo-spheroids [primary PDAC5 cells sorted for SSEA4 (PDAC5 (SSEA4)] and hetero-spheroids [PSC/PDAC5 (SSEA4)] as determined by luciferase assay. PDAC5 (SSEA4) cells alone or mixed with immortalized PSCs at different ratios were grown in 96-well cell repellant plates to form homo- and hetero-spheroids, respectively. After 4 days of incubation, the spheroids were treated with different drugs and incubated for further 3 days as also shown in representative images of spheroids treated with gemcitabine taken on days 4 and 7, **A** left panel). The spheroids were treated with cytotoxic agents gemcitabine (**A**), and oxaliplatin (**B**) as well as c-MET inhibitors, tivantinib (**C**), PHA-665752 (**D**) and crizotinib (**E**) for 3 days and cancer cell proliferation was assessed by luciferase assay. The data represent the mean ± standard error of mean (S.E.M.) of three to five experiments (* and **: the difference between the hetero-spheroid and homo-spheroid treated with same dose of drug was significantly different at *p* < 0.05 and *p* < 0.01, respectively). Abbreviations: PDAC: pancreatic ductal adenocarcinoma; SSEA4, stage specific embryonic antigen-4; PSC, pancreatic stellate cell.

In order to measure PDAC drug response in 3D conditions in the presence or absence of PSCs, homo- and hetero-spheroids were treated with various concentrations of cytotoxic drugs, including gemcitabine and oxaliplatin, as well as c-MET inhibitors, tivantinib, PHA-665752 and crizotinib for 72 h (Figure 6). Crizotinib’s effect could be ascribed to solely MET inhibition, because the expression data in PDAC cells (Supplemental Figure S2) showed that MET expression was much higher compared to anaplastic lymphoma receptor tyrosine kinase (ALK), which had very low levels in these cells. 

All spheroids were much more resistant to cytotoxic drugs compared to monolayer cultures: In PDAC5 (SSEA4) homo-spheroids, gemcitabine concentrations as high as 50 µM decreased the growth only to 55.4%. This is remarkable if we consider that gemcitabine in monolayer cultures reduced the viability of PDAC5 (SSEA4) cells to less than 50% at a much lower dose of 50 nM. In addition, the hetero-spheroids, especially those with 1:1 and 1:2 ratios, were even more gemcitabine resistant compared to homo-spheroids.

The same phenomenon of drug resistance in 3D compared to monolayer cultures was also observed for the other cytotoxic agent, oxaliplatin. Further, the 1:1, 1:2 and 1:4 PSC/PDAC5 (SSEA4) spheroids were more resistant to oxaliplatin at 25 µM compared to homo-spheroids, showing a trend towards a significant difference (p = 0.06 for PSC/PDAC5 (SSEA4) 1:1 at 25 µM compared to PDAC5 (SSEA4)).

Importantly, while hetero-spheroids seemed to be more resistant to cytotoxic agents compared to homo-spheroids, the c-MET inhibitors, especially tivantinib and PHA-665755, were equally effective against homo- and hetero-spheroids (Figure 6C,D).

### 2.7. Effect of Combination of Gemcitabine and c-MET Inhibition in 3D Culture

**Figure 7:**
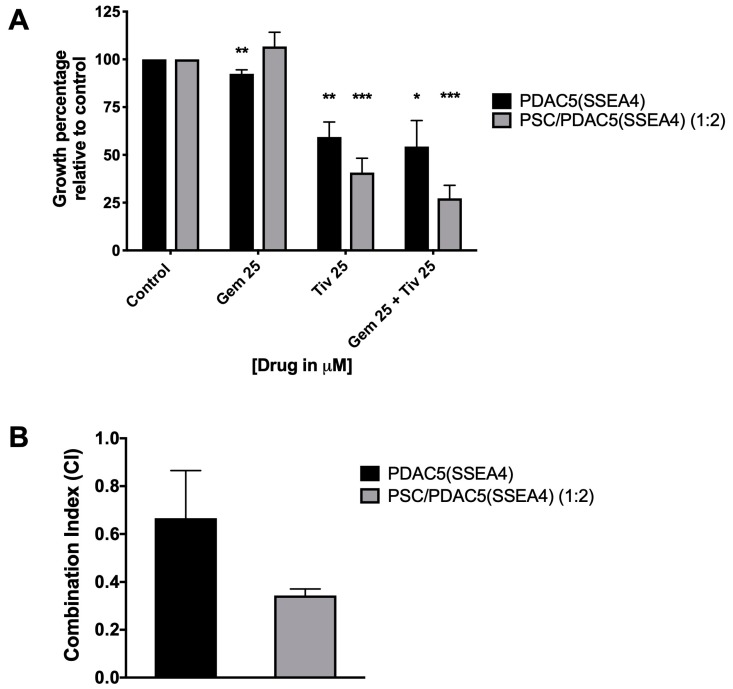
Effect of combination therapy on homo-spheroids [primary PDAC5 cells sorted for SSEA4 (PDAC5 (SSEA4)] and hetero-spheroids [PSC/PDAC5 (SSEA4)] cultures. The homo- and hetero-spheroids were formed as described in the methods. The 3D cultures were then treated either with gemcitabine or tivantinib or a combination of the two drugs for 3 days. The number of viable PDAC5 (SSEA4) cells were determined in spheroids by luciferase assay and were compared to their respective untreated controls. The data represent the mean ± standard error of mean (S.E.M.) of two to four experiments (*, ** and ***: the difference between drug treated and untreated spheroid was significantly different at *p* < 0.05, *p* < 0.01 and *p* < 0.005, respectively) (**A**). Calculation of combination index (CI) with Calcusyn software showed synergism (CI < 0.9) between gemcitabine and tivantinib, especially in PDAC5 (SSEA4) cells (**B**). Abbreviations: PDAC, pancreatic ductal adenocarcinoma; SSEA4, stage specific embryonic antigen-4; PSC, pancreatic stellate cells.

Homo- and hetero-spheroids were treated with a combination of gemcitabine and tivantinib, which emerged as the most active c-MET inhibitor, under the conditions described above. The combination of gemcitabine and tivantinib resulted as more effective compared to single treatments, and this effect was synergistic in the hetero-spheroids (Figure 7).

### 2.8. Growth and Drug Response of PDAC Cells in the Presence of Primary PSCs in 3D Cultures Determined by Luciferase Assay

**Figure 8:**
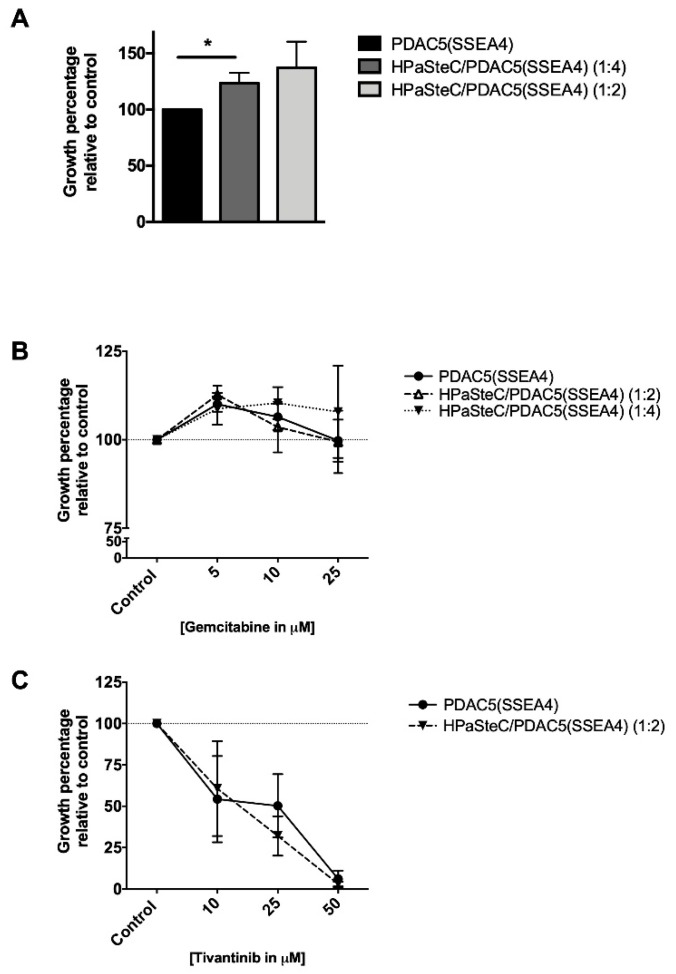
Effect of primary human pancreatic stellate cells (HPaSteC) on proliferation and drug response of primary PDAC5 cells sorted for SSEA4 (PDAC5 (SSEA4)) in 3D culture determined by luciferase assay. PDAC5 (SSEA4) cells alone or mixed with HPaSteC at different ratios were grown in 96-well cell repellant plates as described in Figure 6 and Supplemental Figure S7. HPaSteC cells significantly increased PDAC5 (SSEA4) cell growth in hetero-spheroids (**A**). Homo- and hetero-spheroids were treated with gemcitabine (**B**) or tivantinib (**C**) for 3 days and cancer cell proliferation was assessed by the luciferase assay. The drugs were tested in duplicate wells and the data represent the mean ± standard error of mean (S.E.M.) of three experiments (*: the difference between the hetero-spheroid and homo-spheroid consisting of the same number of cancer cells was considered significantly different at *p* < 0.01). Abbreviations: PDAC, pancreatic ductal adenocarcinoma; SSEA4, stage specific embryonic antigen-4; PSC, pancreatic stellate cells.

In order to confirm the effect of immortalized PSCs on PDAC growth and drug response, we performed additional experiments using primary human pancreatic stellate cells (HPaSteC) in 3D hetero-spheroid cultures. The growth and drug response were examined by luciferase assay. Similar to our observation with immortalized PSCs, HPaSteCs also stimulated PDAC cell growth in spheroids (Figure 8A). Furthermore, PDAC5 (SSEA4) and HPaSteC/PDAC5 (SSEA4) spheroids seemed to be very resistant to gemcitabine (Figure 8B). However, the growth of both homo- and hetero-spheroids was dose-dependently reduced in response to tivantinib (Figure 8C).

### 2.9. Effect of Tivantinib on Tubulin Polymerization in PDAC Cells

Previous data in different cancer cell lines have supported the role of tivantinib in the inhibition of mitosis and impairment of cytoskeleton dynamics [32,33]. Thus, we evaluated whether tivantinib could modulate microtubule stability, using a previously validated “whole cell” methodology for the quantitative analysis of tubulin polymerization levels within the cells [33,34]. PDAC1 and PDAC5 cells were incubated with tivantinib and tubulin fluorescence was assessed by flow cytometry using a specific anti-α-tubulin antibody. In this assay tivantinib did not induce a significant modulation of the fluorescence signal (Supplemental Figure S8). These results suggest that the ability of tivantinib of disrupting microtubules is cell-dependent and does not occur in the primary PDAC cells evaluated in the present study.

## 3. Discussion

In this study, we used 2D and 3D cultures in order to explore the intricate interactions between human PSCs and primary pancreatic ductal adenocarcinoma (PDAC) cells. The PSCs conditioned medium (PCM), when added to monolayer cultures of primary PDAC5 (SSEA4), a subclone of PDAC5 cells resulted in considerable resistance against gemcitabine, associated to increased phospho-c-MET expression. Then, we successfully developed a 3D hetero-spheroid model consisting of the same primary PDAC cells and PSCs, in which we showed that the presence of PSCs significantly enhances the growth and gemcitabine resistance of PDAC5 (SSEA4) cells. However, c-MET inhibitors were equally effective against PDAC cells either in monolayer culture in the presence of PCM or in PSC/PDAC hetero-spheroids. 

The mutual interaction between stromal components and PDAC cells has been the focus of several recent studies [13,31,35,36,37]. It has lately been suggested that hepatocyte growth factor (HGF) secreted by PSCs and the presence of HGF’s receptor c-MET on PDAC cells, may play a major role in the tumor promoting effect of PSCs on PDAC cells [29,31,35,36,37,38]. Moreover, PDAC cell lines have been orthotopically co-injected with PSCs in mice and it has been observed that tumor size and progression was significantly higher compared to mice that had been injected with PDAC cells alone [20,39]. In addition, HGF has been shown to induce chemoresistance in different cancer cells [40,41] and serum HGF levels have been correlated with prognosis and response to targeted therapies in colorectal cancer patients [42].

We observed that PDAC5 (SSEA4) cells showed enhanced phospho-c-MET expression when incubated with PCM. Based on these findings and our previous findings that c-MET is a valuable target in PDAC cells driven by this oncogenic pathway [27], these cells were used for monolayer and 3D experiments.

We further observed that HGF secreted by PSCs was increased 3.5 times when these cells were stimulated with PDAC conditioned medium. Several signaling molecules secreted by PDAC cells could stimulate PSCs to secrete growth factors. For instance, galectin-3 [35], sonic hedgehog [31] and mutated KRAS [43] produced by PDAC cells have been implicated to mediate this effect. This phenomenon is extremely interesting since it shows that there is a reciprocal stimulatory interaction between cancer and stromal cells, as corroborated by several recent findings [31,37,43]. HGF is among the most important growth factors that are secreted by PSCs in response to the stimulus received by PDAC cell and can, in turn, induce growth and drug resistance in cancer cells [30,31].

In monolayer culture, the presence of PCM rendered the PDAC cells highly resistant to gemcitabine; concentrations that stopped the growth of cancer cells by more than 75% in normal condition, were indeed almost ineffective against PDAC cells in the presence of PCM. These results are in agreement with previous investigations reporting that PSC conditioned medium induced gemcitabine resistance of the PDAC cell lines Panc-1 and BxPC3 [21]. However, in the present study, we also noticed that, under the same circumstances, several c-MET inhibitors were equally effective in the presence or absence of PCM. This observation strongly supports the notion that HGF is a principal component of PCM that causes cytotoxic drug resistance in cancer cells, which is consistent with previous studies [30,31,44]. Moreover, a recent report showed that an HGF neutralizing antibody and a small c-MET inhibitor combined with gemcitabine greatly reduced tumor size in an orthotopic mouse model of PDAC [38]. Of note, two of the c-MET inhibitors evaluated in our study, crizotinib and tivantinib, are already used in the clinical setting for different tumor types [45] and a phase I trial of gemcitabine combined with tivantinib, which emerged as the most active compound in our 3D models, showed good tolerability and early signs of antitumor activity, warranting further development of this combination in several solid tumors, including PDAC [46]. However, the negative results of the “Tivantinib for second-line treatment of MET-high, advanced hepatocellular carcinoma” (METIV-HCC) phase III randomized trial, showing that tivantinib did not improve overall survival compared with placebo in patients with c-MET-high advanced hepatocellular carcinoma, suggest that additional studies are needed to find biomarkers to identify the subsets of patients more sensitive to c-MET inhibition [47]. For instance, our previous data showed that the synergistic interaction of tivantinib and gemcitabine was not associated with the increase in the accumulation of gemcitabine-nucleotides, as observed for crizotinib-gemcitabine. Furthermore, no modulation of cytidine deaminase activity was determined by direct interaction with tivantinib. Conversely, we observed a significant inhibition of tubulin polymerization both after tivantinib and gemcitabine-tivantinib exposure in other cell lines [48].

We reckon that these complex molecular mechanisms should be further investigated in more appropriate preclinical models. Regarding this issue, it has been recently shown by several lines of evidence that 3D models possess key characteristics that provide biologically relevant conditions and hence constitute an improved platform for pharmacological studies [22,24,49].

Indeed, around 95% of anticancer agents that prove effective in the existing preclinical models, eventually fail in different stages of clinical assessments. This problem has raised serious doubt about the simple and very reductionist monolayer culture systems that do not incorporate the complexity of cancer cell microenvironment, including the reciprocal interaction with stromal cells in a 3D space [24]. Furthermore, in light of the guiding principles of animal research like the “3Rs”, which stand for replacement, reduction and refinement, in vitro models such as spheroids could be an appropriate alternative to replace the use of animals and reduce costs to test pharmacological anticancer drugs, such as patient-derived xenografts (PDX) and genetically engineered mouse models [50].

Hence, in order to shed further light on how PSCs and PDACs interact with each other, we set up a 3D model incorporating both cell types. Our spheroids grew steadily in commercially available 96-well cell repellant plates and the expression of Fluc in PDAC cells allowed reliable measurement of cancer cell growth by luciferase assay in these 3D cultures. Other investigators have used PDAC cell lines in a co-culture 3D system together with PSCs [51]. However, to our knowledge, this is the first report on the use of primary PDAC cells in a hetero-spheroid model. 

Early passages of primary PDAC cells better mimic the genetic characteristics of the disease and might be better predictors of anticancer drug activity [27]. Moreover, we observed that PDAC5 (SSEA4) cells in both homo-and hetero-spheroids were barely responsive to concentrations of up to 10 µM of the cytotoxic agents gemcitabine and oxaliplatin, i.e., more than 200 times higher than inhibitory concentration 50 (IC_50_) values in monolayer culture. This extreme drug resistance has been also reported by other investigators in cancer spheroid models [52] and demonstrates once again that 3D cultures more closely mimic the real in vivo conditions in which the PDAC tumor is usually inherently resistant to cytotoxic agents [22]. Remarkably, PSC/PDAC5 (SSEA4) hetero-spheroids were even more resistant to gemcitabine compared to PDAC5 (SSEA4) homo-spheroids. However, the c-MET inhibitors tivantinib, PHA-665755 and crizotinib affected homo- and hetero-spheroids equally. This further suggests that c-MET inhibitors represent a useful asset in control of PDAC cell growth in its microenvironment that normally contains activated PSCs [29].

When primary human PSCs (HPaSteC) cells were used for spheroid formation, the observed drug responses were very similar to the results when immortalized PSCs were used in the 3D model. This confirms that the observed tumor-promoting effect is not limited to immortalized PSCs.

Aside from drug resistance, it would be interesting to investigate the resistance to radiotherapy using co-cultured spheroids. PSCs were shown to mediate resistance to radiation treatment to PDAC cells via activation of β1-integrin signaling [53]. Previous studies have also demonstrated the use of spheroids in regards to radioresistance in human gliomas, albeit without consideration for the potential role the tumor microenvironment could play [54]. In order to combat the drug- and radioresistance mediated by PSCs in PDAC, it would therefore be also crucial to investigate possible combinations of pharmacological and/or radiotherapeutic strategies, for example through combinations of radiosensitizing and cytotoxic agents with radiation treatment. 

Finally, another intriguing finding in this study was the structural reorganization that took place in hetero-spheroids revealed by confocal microscopy. Immortalized PSCs were found to move to the periphery in small clusters on the border of the formed PDAC spheroids within 24–48 h after seeding. A possible explanation is the phenomenon of ‘leading cells’, which lead cancer cells and stimulate invasion. Koikawa and colleagues recently showed that PDAC cells follow PSCs in co-cultures on a collagen matrix [55]. In our hetero-spheroids, it is possible that PSCs were migrating to the periphery, while in an ultra-low attachment surface the migration remained limited.

## 4. Materials and Methods 

### 4.1. Cell Culture 

Primary human pancreatic ductal adenocarcinoma cells (PDAC1, PDAC2, PDAC3 and PDAC5) were isolated from patients undergoing pancreaticoduodenectomy in Pisa University Hospital, according to a protocol approved by the Ethics Committee at the same university hospital (Pisa, Italy, date of approval: July 3, 2013 (file number 3909)) [56].The resulting primary cultures were transduced with lentiviral vectors encoding firefly luciferase and CFP, as described previously [56,57]. PDAC5 cells were sorted for stage-specific embryonic antigen-4 (SSEA4) positive cells by flow cytometry to generate the PDAC5 (SSEA4) subclone [58]. 

A human immortalized PSC line expressing GFP was prepared as described earlier [59]. Primary human PSCs were purchased from ScienCell Research Laboratories (Carlsbad, CA USA) and used for comparison of the findings obtained with immortalized PSCs in luciferase assay.

For monolayer cultures, all cells were grown in complete growth medium including Roswell Park Memorial Institute-1640 medium (RPMI-1640, Lonza, BioWhittaker®, Basel, Switzerland) supplemented with 10% heat-inactivated fetal bovine serum (FBS, Biowest, Nuaillé, France) and 1% penicillin/streptomycin (P/S, Lonza, BioWhittaker®, Basel, Switzerland) at 37 °C in the presence of 5% CO_2_. The cells were subcultured twice a week using trypsin/ethylenediaminetetraacetic acid (EDTA, Lonza, BioWhittaker®, Basel, Switzerland) at a confluence of approximately 80%. Immortalized PSCs were incubated in 1 µg/mL puromycin (SigmaAldrich, St. Louis, MO, USA) after thawing for selection of GFP positive cells. For 3D experiments, the cells were adapted to serum fee conditions as described below.

For spheroid cultures, the cells were first adapted to serum-free medium. The concentration of FBS was decreased stepwise over the course of two weeks. FBS was substituted by a growth factor cocktail consisting of 20 ng/mL EGF, insulin (10 µg/mL), transferrin (5.5 µg/mL), selenium (6.7 ng/mL) (ThermoFischer Scientific, Gibco, Cat#41400-045, Waltham, MA, USA) and Glutamax (2 mM) (ThermoFischer Scientific, Gibco, REF#35050-038, Waltham, MA, USA).

### 4.2. Enzyme-Linked Immunosorbent Assay (ELISA) of Phospho- and Total c-MET

The binding of HGF with c-MET induces receptor dimerization, which results in trans-phosphorylation of the two tyrosine residues Y1234 and Y1235 within the catalytic domain, allowing for the recruitment of signal-relay molecules [60]. Therefore, the expression of phospho-c-MET was further studied by a specific ELISA assay for these phosphorylation sites. Total c-MET expression was also analyzed by another specific ELISA assay. Cells were plated for 24 h at a density of 100,000 cells/mL.

After protein extraction from PDAC1, 2, 3, 5 and PDAC5 (SSEA4) cell pellets, c-MET phosphorylation at tyrosine residues 1230, 1234, and 1235 (i.e., c-MET autophosphorylation sites [pYpYpY1230/34/35]) was evaluated with the ELISA assay #KHO0281 (Thermo Fisher Scientific, Waltham, Massachusetts, USA), while total c-MET was evaluated with the ELISA assay #KHO0251 (Thermo Fisher Scientific, Waltham, MA, USA), as described previously [33]. Values obtained (using 10 mg/mL of protein lysates) in PDAC cells were calculated using standard curves, which were run with each specific assay using 100, 50, 25, 12.5, 6.25, 3.12, and 1.6 units/mL of standard human phosphorylated c-MET [pYpYpY1230/1234/1235], and 50, 25, 12.5, 6.25, 3.12, and 1.6 ng/mL of standard recombinant human c-MET, respectively.

Furthermore, in order to assess the phosphorylation induced by HGF incubation, PDAC1, PDAC5 and PDAC5 (SSEA4) cells were seeded in six-well plates at a density of 100,000 cells/mL. After 24 h incubation, HGF was added at 20 and 60 pg/mL and the cells were further incubated for 24 h. The protein was extracted with the extraction buffer provided with the kit and total c-MET and phospho-c-MET were analyzed by ELISA as described above.

### 4.3. Immunofluorescence Staining 

PDAC1, 2, 3, 5 and PDAC5 (SSEA4) cells were seeded in an Eight-Chamber-Slides System (ThermoFischer Scientific, Lab-Tek, Waltham, MA, USA) at a density of 100,000 cells/mL and grown for 24 h (2 mL of cell suspension in each well). Afterward, one third of the complete growth medium was substituted with PSC conditioned medium and the cells were incubated for further 24 h. The cells were fixed and stained with specific monoclonal rabbit anti-human c-MET and anti-phospho-Y1003-c-MET antibodies (1:200 dilution; Santa Cruz Biotechnology, Dallas, TX, USA), as described previously [27].

The quantification of c-MET and phospho-c-MET staining was performed by the immunofluorescence (IF) assay. After fixation with 4% paraformaldehyde (PFA), slides were rinsed in PBS 1X for 10 min. After washing, the fluorescent secondary antibody was applied (1:50; 30 min at RT in darkness; Anti-rabbit IgG Fab2 AlexaFluor 488, and Anti-rabbit IgG Fab2 AlexaFluor 555 (Cell Signaling Technology, Danvers, MA, USA). The nuclei counterstaining was performed using a special fluorescence antifade containing 4′,6-diamidino-2-phenylindole (DAPI, ProLong® Gold Antifade Reagent with DAPI #8961, (Cell Signaling Technology, Danvers, MA, USA).

Samples were stored at 4 °C until analysis. The visualization and quantification were performed using a confocal microscope (Axio vert 200, Carl Zeiss Microscopy, Jena, Germany) and its dedicated software for image acquisition and digital imaging process (AxioVision version 4.2.3.1, Carl Zeiss Microscopy, Jena, Germany). The images were acquired at 40× magnification, using the same exposure time and laser intensity. Six different images (DAPI, Green, Red, Merge (M), Bright field (BF) and BF + M) were obtained for each field, as reported in the Figure 1.

### 4.4. Stimulation of PSCs with PDAC Medium 

PSCs were stimulated with PDAC conditioned medium for 3 days. This was critical in order to obtain “stimulated” PSC conditioned medium (PCM, Supplemental Figure S3). Because the PDAC5 (SSEA4) subclone had the highest baseline phospho-c-MET expression we selected these cells to prepare conditioned medium, as follows: cells were grown in T75 cm^2^ flasks to a confluence of 90%. Afterward, the cells were washed and the medium was aspirated and substituted with RPMI-1640 medium (without any further supplements) and cells were incubated for further 72 h. Medium was then collected and centrifuged at 500 × g for 7 min and the supernatant was collected and stored at −20 °C. 

To obtain stimulated PCM, serum-free adapted PSCs (7 × 10^6^ cells) were seeded in a 175 cm^2^ culture flasks with one third of the PDAC5 (SSEA4) conditioned medium and two-thirds of serum free medium. After 72 h of stimulation at 37 °C, the medium was removed, the cells were washed and supplemented with RPMI-1640 and incubated for additional 72 h. Finally, conditioned medium of stimulated PSCs was collected and centrifuged at 500 × g for 10 min. The supernatant was transferred into Amicon Ultra-15 Centrifugal Units, MWCO 3K (Merck Millipore, UFC90038, Burlington, MA, USA) and centrifuged at 3500 × g for 60 min at 4 °C to prepare a 24× concentrated PCM. This “stimulated” PCM was stored at −20 °C till analysis.

### 4.5. Measurement of Hepatocyte Growth Factor in PSC Conditioned Medium and Serum Samples

To quantify the hepatocyte growth factor (HGF) levels secreted by stimulated and non-stimulated (baseline) PSCs, we performed a quantitative human HGF Enzyme-linked immunosorbent assay (ELISA) by using a human HGF Quantikine ELISA kit (R&D Systems, Minneapolis, MN, USA) according to the manufacturer’s instructions. Optical density was measured at 450 nm with a correction wavelength at 540 nm using the BioTek plate reader (BioTek Intruments Inc., Winooski, VT, USA). Since a recent study showed a correlation between high level of circulating cytokines and unresponsiveness in PDAC patients [61], we used the same ELISA kit to perform an exploratory analysis in the serum of 10 patients with metastatic PDAC who were treated with gemcitabine [62]. The study was approved by the ethics committee of the University of Pisa (file number/approval 3773/2012)), and written informed consent was obtained from all the patients enrolled in the study.

### 4.6. Sulforhodamine B Assay in Monolayer Culture 

To determine drug-induced cytotoxicity in monolayer culture, the sulforhodamine B (SRB) assay was performed using PDAC5 and PDAC5 (SSEA4) cells grown in RPMI containing 10% FBS as described earlier [63]. One hundred µl of cell suspension at a density of 3000 to 5000 cells/mL was added to each well of 96-well flat-bottom microplates. After incubation at 37 °C overnight, 100 µL of test drugs at different concentrations were added in triplicate, while 100 µL medium was added to control cells. The above-described concentrated stimulated PCM was added to the proper wells in a 1:10 dilution right before the addition of the drugs. The plates were then incubated at 37 °C for 72 h, after which the cells were fixed with 25 µL of 50% cold trichloroacetic acid added to each well for 1 h at 4 °C. After fixation, the plates were washed five times with distilled water. They were then left to dry overnight and 50 µL of SRB staining solution (SRB 0.4% (w/v) dissolved in acetic acid 0.1%) was added per well. After 15 min, the plates were emptied, washed four times with 1% acetic acid (SigmaAldrich, St. Louis, MO, USA), and again left to dry. Finally, 150 µL of Tris base solution (10 mM, Merck, Darmstadt, Germany) was added to each well and the optical densities were measured at 490 nm using a BioTek microplate reader (BioTek Instruments Inc., Winooski, VT, USA). For measurement of tivantinib effect against PDAC1-3 cells, the experiments were performed as described above with the exception that tivantinib was incubated with cells for 48 h.

### 4.7. Growth of Spheroids in 96-Well Cell Repellant Plates 

PDAC5 (SSEA4) cells as well as immortalized and primary human PSCs were first adapted to serum-free conditions in the course of 2 weeks as described earlier before making spheroids. When the cells were fully adapted to serum-free conditions, homo- and hetero-spheroids, consisting of cancer cells alone or cancer and PSCs together, respectively, were made in CELLSTAR® 96-well cell repellent U-bottom plates (Greiner Bio-One, Cat No. 650970, Kremsmünster, Austria). Single cell solutions of PSCs and PDAC5 (SSEA4) were prepared and then mixed at different ratios (1:1, 1:2, 1:4 and 1:8, respectively) in 15 or 50-ml Falcon tubes. Two hundred and ten µL of different cell suspensions were then added to each well and left to incubate for 96 h at 37 °C in 5.0% CO_2_, during which spheroids were formed. The number of PDAC5 (SSEA4) cells in each well were 20,000, while the number of PSCs varied from 0 in homo-spheroids to 2500–20,000 in hetero-spheroids according to the ratio of PSC to PDAC cells (1:8 to 1:1, respectively). After 4 days, 140 µL of medium was replaced by media containing various concentrations of drugs. This step was carefully performed by tilting the plate at 45 degrees and carefully placing the pipette tips to the side of the well in order not to disrupt the spheroid structure.

To assess the drug resistance in homo- and hetero-spheroids, we selected two cytotoxic drugs which are commonly administered to PDAC patients in the clinical setting, such as gemcitabine and oxaliplatin. Furthermore, c-MET inhibitors, such as tivantinib, crizotinib and PHA-665752 (Selleck Chemicals, Houston, TX, USA) were used. Drug-treated spheroids were then incubated for 72 h at 37 °C and 5% CO2. Spheroid bright field images were taken daily with a Leica DM3000 B microscope (Leica Microsystems, Wetzlar, Germany). These spheroids were also used for the firefly luciferase assay and confocal imaging, as described below.

### 4.8. Firefly Luciferase Assay 

PDAC5 (SSEA4) which expressed Fluc provided the basis for quantification of the effect of the various drugs on cancer cells in homo- and hetero-spheroids, by measurement of luminescence. In this assay, a higher luminescence signal corresponds to a higher signal related to viable PDAC5 (SSEA4) cells in spheroids. After drug treatment of spheroids for 3 days, 100 µL of medium was removed from each well. As described above, this step was carefully performed by tilting the plate at 45 degrees and gently placing the pipette tips to the side of the well. Potassium D-luciferin (Gold Biotechnology, Cat. No. LUCK-1G, St. Louis, MO, USA) was diluted 1:5 in phosphate buffered saline (PBS) and added to each well to obtain a final concentration of 8.2 µM. The plates were incubated for 1 h at 37 °C. Luminescence was measured with the BioTek plate reader (BioTek Instruments Inc., Winooski, VT, USA).

The pharmacological interaction of gemcitabine and tivantinib was assessed using the multiple drug effect analysis based on the methods described by Chou and collaborators in which a Combination Index (CI) CI < 0.9 means synergism; CI = 0.9–1.1 means additive interaction; CI > 1.1 means antagonism, as reported previously [64]. The data were processed by the Calcusyn Software (Biosoft, Cambridge, UK) which calculates the CI of the combination based on the effect of the growth inhibition caused by the drugs alone relative to the effect produced by the combination.

### 4.9. Confocal Microscopy

Three-dimensional live spheroid imaging was performed on a home-built setup based on an Axiovert 200 microscope body (Carl Zeiss Microscopy, Jena, Germany). Confocal imaging was achieved by means of a spinning disk unit (CSU-X1, Yokogawa, Musashino, Tokyo, Japan). The confocal image was acquired on an emCCD camera (iXon 897, Andor). IQ-software (Andor) was used for basic setup-control and data acquisition. Illumination of CFP- and GFP-labeled spheroids was performed with two different lasers of wavelengths 405 (CrystaLaser, Reno, NV, USA) and 488 nm (Coherent Inc., Santa Clara, CA, USA). Accurately controlled excitation intensity and excitation timing were achieved using an acousto-optic tunable filter (AA Optoelectronics, Orsay, France). Light was coupled into the confocal spinning-disk unit by means of a polarization maintaining single-mode fiber (OZ Optics, Ottawa, Canada). The fluorescent signal was collected by a 10×/0.3 air objective (Carl Zeiss Microscopy, Jena, Germany). Three-dimensional images of PDAC/PSC spheroids were obtained by imaging 100 µm z-stacks (every 1 µm) using a piezo system (Physik Instrumente, Karlsurhe, Germany).

### 4.10. Microtubule Stabilization Analysis

PDAC1, and PDAC5 cells were seeded in six-well plates (1 × 10^5^ cells/well) and exposed for 24 h with either vehicle or tivantinib 2.5 and 8.1 µM (in PDAC1 and PDAC5 cells, respectively). Then the cells were trypsinized, harvested, and microtubules stabilized in MicroTubule Stabilizing Buffer (80 mM Pipes [pH 6.8], 1 mM MgCl2, 5 mM EDTA, and 0.5% Triton X-100), as described previously [34]. Staining was performed with anti-tubulin-Fitc conjugated antibody (1:50, CA #8058, Cell Signaling Technology, Danvers, MA, USA) and analyzed by flow cytometry on a FACSCalibur (Becton Dickinson, Franklin Lakes, USA).

### 4.11. Statistical Analysis 

In comparison of two un-paired groups such as PDAC cells in the presence or absence of PCM (Figure 1 and Figure 3), samples were analyzed with unpaired t-test using the GraphPad Prism version 7 software (GraphPad Software, San Diego, CA, USA). Whenever several groups were compared (Figure 5, Figure 6, Figure 7 and Figure 8), one-way analysis of variance (ANOVA) with Fisher’s Least Significant Difference (LSD) post hoc test was applied with the same software.

## 5. Conclusions

The findings of this study show that there is a reciprocal interaction between PDAC cells and an important component of their microenvironment, PSCs. In the context of this pathologic liaison, cancer cells stimulate PSCs to secrete growth factors such as HGF, and in turn HGF and probably other factors secreted by these stromal cells induce proliferation and drug resistance in PDAC cells. We also establish for the first time a 3D model encompassing primary human PDAC cells and PSCs. This method, which could be used for high throughput pharmacological screenings, shows that c-MET inhibitors, different from cytotoxic agents such as gemcitabine and oxaliplatin, can be effective against cancer cell growth in the presence of PSCs. Hence, the findings of this study present further evidence on the role of PSCs in providing growth support and induction of drug resistance for PDAC cells. This report also establishes a biologically relevant new model that takes into account the contribution of PDAC microenvironment and represents an important tool for a more realistic pharmacological assessment of anticancer as well as anti-stroma-directed therapies.

## Supplementary Materials

The following are available online at https://www.mdpi.com/2072-6694/11/5/638/s1, **Figure S1:** Kaplan–Meier (KM) curve for MET mRNA expression. Thirty-two samples lacked survival data and were omitted from the analysis. The online platform R2 (R2.amc.nl) enabled us to perform the KM survival curve. The pancreatic cancer dataset (TCGA data) with a median cutoff was selected (left panel). Graph of MET gene expression sorted from the lowest to the highest labeled by event (right panel). **Figure S2:** Gene expression of MET and ALK in PDAC1-5 primary cultures. MET mRNA expression is significantly up-regulated compared to ALK expression (p = 0.03). **Figure S3:** Expression of phospho-c-MET in human primary PDAC cells. Human primary PDAC cells isolated from PDAC patients (PDAC1, 2, 3, 5 and PDAC5(SSEA4)) were grown in culture plates. Total proteins were extracted from PDAC cells and subjected to analysis by ELISA assay specific for phosphorylated tyrosine residues 1230, 1234, and 1235 as well as total c-MET. Standard curves of phospho-c-MET (**A**) and total c-MET (**B**) as well as base line c-MET expression levels (**C**) are shown. **Figure S4:** Schematic description of stimulation of PSCs with PDAC medium, in order to obtain “stimulated” PSC conditioned medium (PCM). Incubation of PDAC cells with PCM resulted in gemcitabine resistance, **Figure S5:** Representative image showing 84 single homo-(PDAC5(SSEA4)) or hetero-spheroids (PSC/PDAC5(SSEA4)) formed in each well of a 96-well cell repellant plate, 96 h after seeding cells. For the formation of homo-spheroids, we used 20,000 PDAC5 (SSEA4) cells, while the hetero-spheroids were formed using 20,000 PDAC5 (SSEA4) cells growing in co-culture with 10,000 or 20,000 PSC cells. **Figure S6:** Three-dimensional reconstructions of PSC/PDAC5 (SSEA4) spheroids at 24 h (**A**) and 48 h (**B**) after seeding. The 3D reconstruction was created using ImageJ (NIH) from confocal z-stacks (100 μm with 1 μm steps). These figures are z-stack movies embedded in .ppt to show the three-dimensional rendering of the representative spheroids in Figure 3C. **Figure S7:** Schematic representation of formation of spheroids and the luciferase assay, which was used to evaluate the growth of the co-cultured PDAC spheroid models. **Figure S8:** Effect of tivantinib on microtubule stabilization and growth in PDAC cells. PDAC1 and PDAC5 cells were seeded in six-well plates and exposed for 24 h with either vehicle or tivantinib 2.5 and 8.1 µM (in PDAC1 and PDAC5 cells, respectively). Then the cells were trypsinized, harvested, and microtubules stabilized in MicroTubule Stabilizing Buffer. Staining was performed with anti-tubulin-Fitc conjugated antibody and the cells were analyzed by flow cytometry. Tivantinib did not induce a significant modulation of the fluorescence signal (**A**). PDAC1-3 cells were grown in 96-well plates in monolayer culture and after being exposed to tivantinib for 48 h, their growth was measured by SRB assay as explained in Figure 3 (**B**). **Figure S9:** Measurement of serum HGF levels in PDAC patients. The concentration of HGF was measured in the serum of patients with metastatic PDAC who were treated with gemcitabine. Serum samples from five patients who achieved stable disease (SD) and five patients who underwent progression at restaging of disease (PD) were measured. HGF levels in PD patients was higher than SD subjects, however this difference did not reach statistical significance.
