# Supplementary material for: Role of c-MET Inhibitors in Overcoming Drug Resistance in Spheroid Models of Primary Human Pancreatic Cancer and Stellate Cells
Source: Cancers (Basel). 2019 May 8;11(5):638. doi: 10.3390/cancers11050638 (PMC6562408; doi:10.3390/cancers11050638)
Supplement: Supplementary file 1 [file cancers-11-00638-s001.pdf]

Supplemental Methods

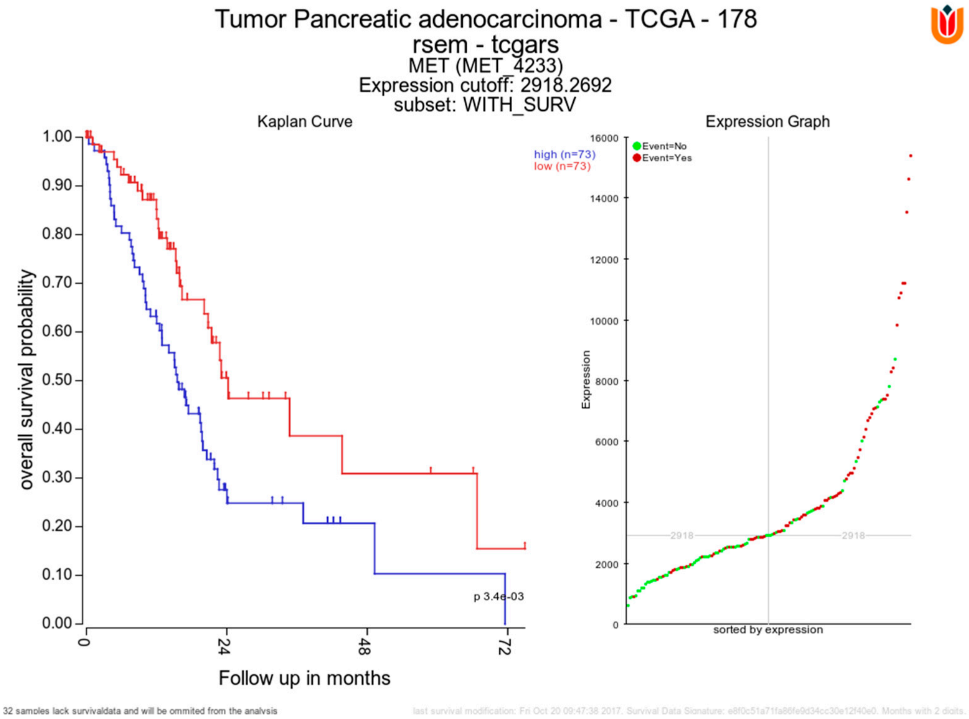

Figure S1. Kaplan-Meier (KM) curve for MET mRNA expression. Thirty-two samples lacked survival data and were omitted from the analysis. The online platform R2 (R2.amc.nl) enabled us to perform the KM survival curve. The pancreatic cancer dataset (TCGA data) with a median cutoff was selected (left panel). Graph of MET gene expression sorted from the lowest to the highest labeled by event (right panel).

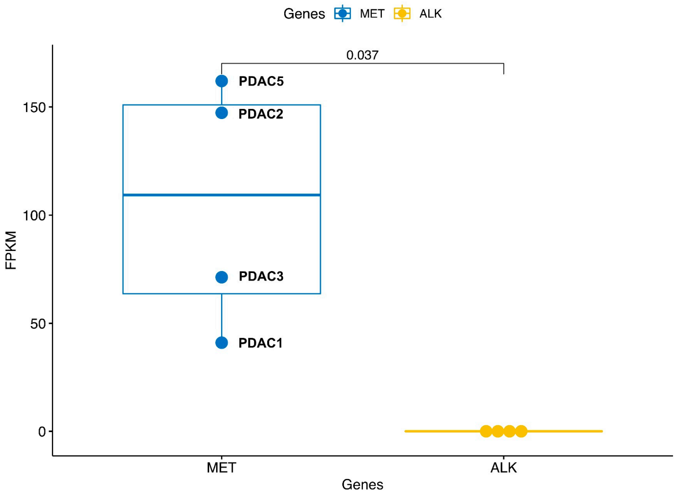

Figure S2. Gene expression of MET and anaplastic lymphoma receptor tyrosine kinase (ALK) in pancreatic ductal adenocarcinoma (PDAC) 1-5 primary cultures. MET mRNA expression is significantly up-regulated compared to ALK expression (p = 0.03).

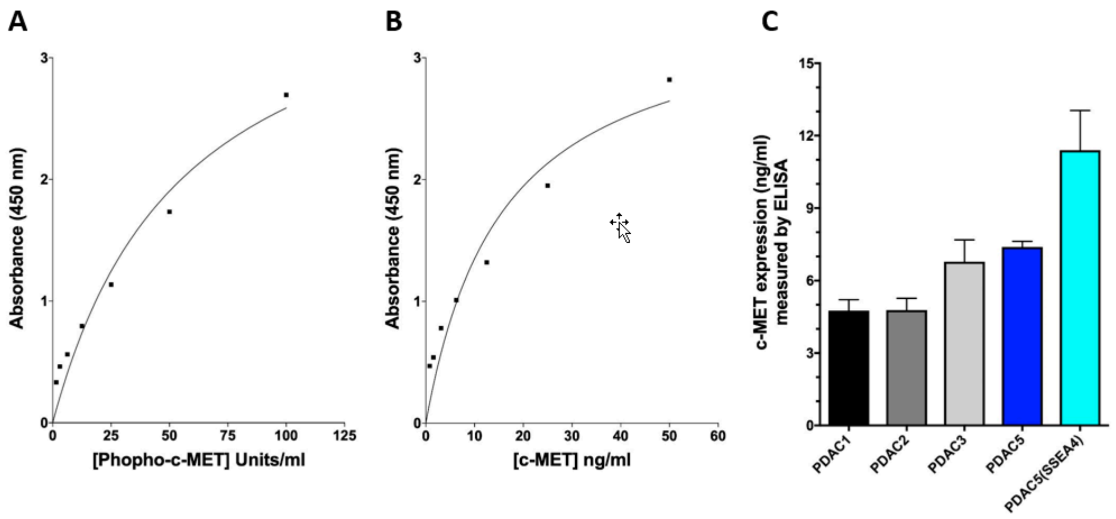

Figure S3. Expression of phospho-c-MET in human primary pancreatic ductal adenocarcinoma (PDAC) cells. Human primary PDAC cells isolated from PDAC patients (PDAC1, 2, 3, 5 and PDAC5(SSEA4)) were grown in culture plates. Total proteins were extracted from PDAC cells and subjected to analysis by ELISA assay specific for phosphorylated tyrosine residues 1230, 1234, and 1235 as well as total c-MET. Standard curves of phospho-c-MET (**A**) and total c-MET (**B**) as well as base line c-MET expression levels (**C**) are shown.

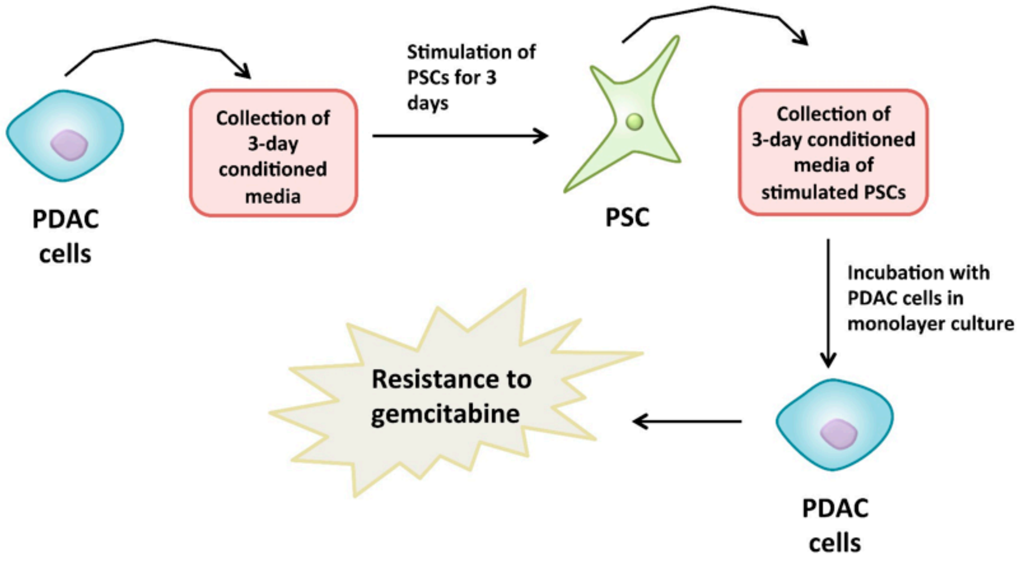

Figure S4. Schematic description of stimulation of pancreatic stellate cells (PSCs) with PDAC medium, in order to obtain "stimulated" PSC conditioned medium (PCM). Incubation of PDAC cells with PCM resulted in gemcitabine resistance.

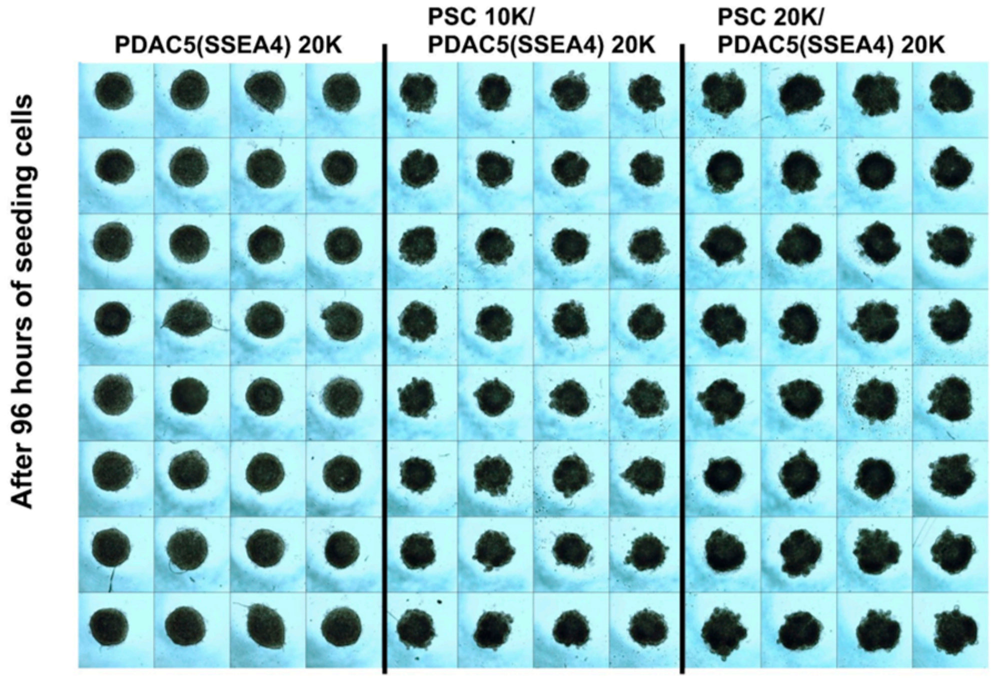

Figure S5. Representative image showing 84 single homo-(PDAC5(SSEA4)) or hetero-spheroids (PSC/PDAC5(SSEA4)) formed in each well of a 96-well cell repellant plate, 96 hours after seeding cells. For the formation of homo-spheroids, we used 20,000 PDAC5(SSEA4) cells, while the hetero-spheroids were formed using 20,000 PDAC5(SSEA4) cells growing in co-culture with 10,000 or 20,000 PSC cells.

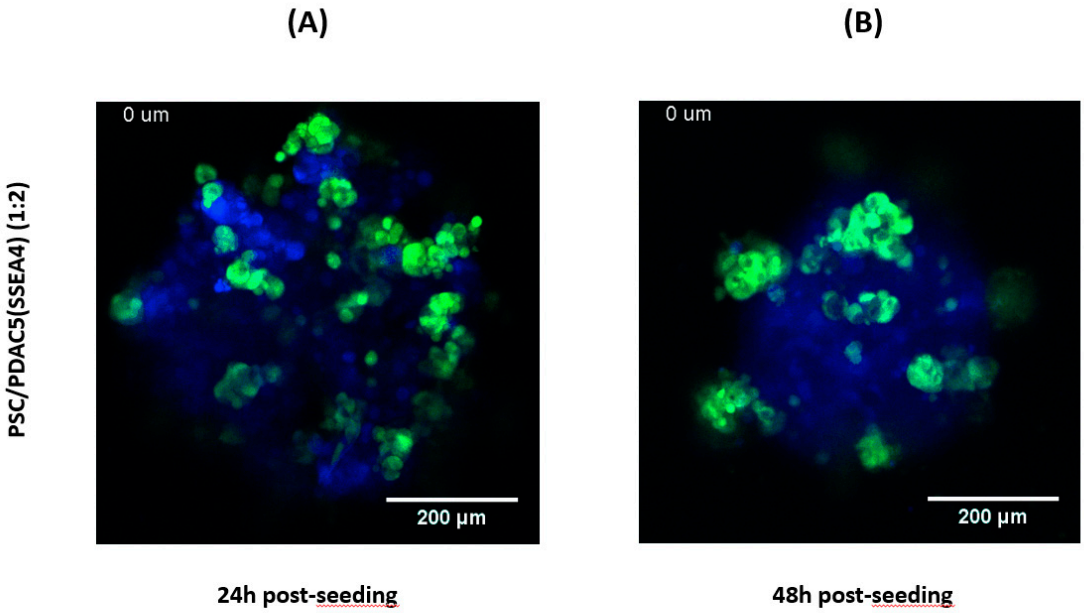

Figure S6. Three-dimensional reconstructions of PSC/PDAC5(SSEA4) spheroids at 24 hours (**A**) and 48 hours (**B**) after seeding. The 3D reconstruction was created using ImageJ (NIH) from confocal z-stacks (100 μm with 1 μm steps). These figures are z-stack movies embedded in .ppt to show the three-dimensional rendering of the representative spheroids in Figure 3C.

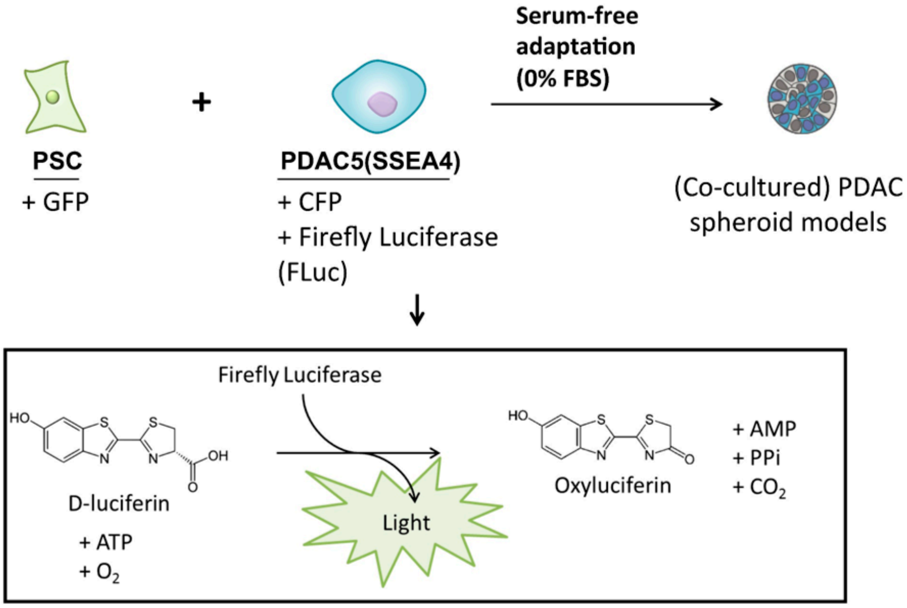

Figure S7. Schematic representation of formation of spheroids and the luciferase assay, which was used to evaluate the growth of the co-cultured PDAC spheroid models.

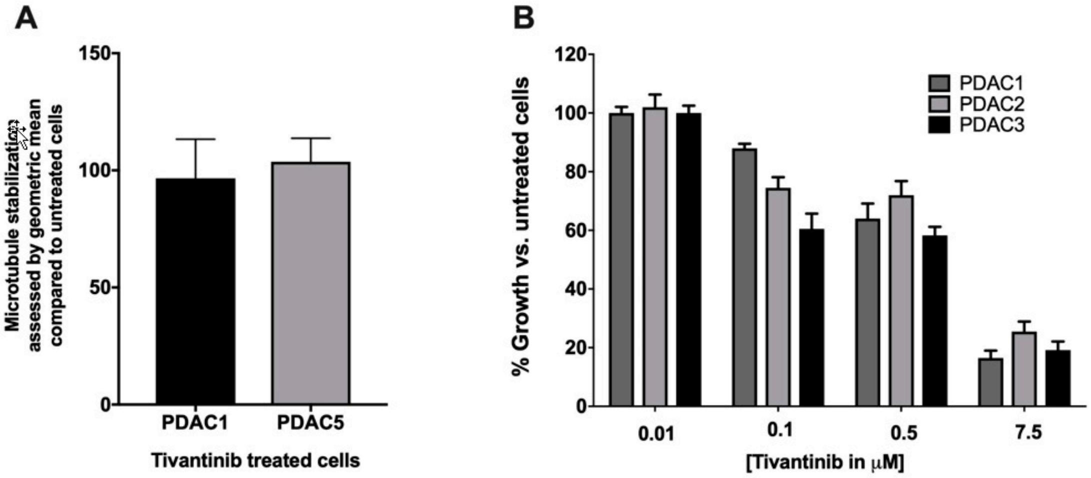

Figure S8. Effect of tivantinib on microtubule stabilization and growth in PDAC cells. PDAC1 and PDAC5 cells were seeded in six-well plates and exposed for 24 hours with either vehicle or tivantinib 2.5 and 8.1 μM (in PDAC1 and PDAC5 cells, respectively). Then the cells were trypsinized, harvested, and microtubules stabilized in MicroTubule Stabilizing Buffer. Staining was performed with anti-tubulin-Fitc conjugated antibody and the cells were analyzed by flow cytometry. Tivantinib did not induce a significant modulation of the fluorescence signal (**A**). PDAC1-3 cells were grown in 96-well plates in monolayer culture and after being exposed to tivantinib for 48 hours, their growth was measured by SRB assay as explained in Figure 3 (**B**).

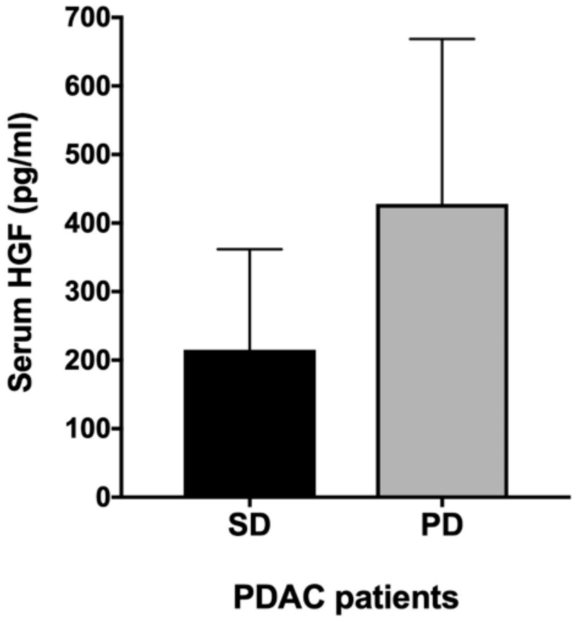

Figure S9. Measurement of serum hepatocyte growth factor (HGF) levels in PDAC patients. The concentration of HGF was measured in the serum of patients with metastatic PDAC who were treated with gemcitabine. Serum samples from five patients who achieved stable disease (SD) and five patients who underwent progression at restaging of disease (PD) were measured. HGF levels in PD patients was higher than SD subjects, however this difference did not reach statistical significance.

Flow Cytometric Analysis and Cell Sorting of PDAC5/SSEA4 Cells

Stage-specific embryonic antigen-4 (SSEA-4) is a glycosphingolipid, which is reported to be expressed in several malignancies. SSEA-4 overexpression is associated with poor prognosis or progression of lung, breast and pancreatic cancer [1,2]. Afrikanova and colleagues identified a subpopulation of human pancreas cells positive for SSEA4, which were expressed as ductal, pancreatic progenitor and stem cell protein markers [3]. Therefore we hypothesized that the subpopulation of the cells expressing SSEA4 might be more representative for the presence of pancreatic stem cells and might be more aggressive than SSEA4 negative cells. The PDAC5 cells were stained with SSEA4 mouse monoclonal antibody (V450 anti-SSEA-4, Clone MC813-70, Becton Dickinson (BD) Horizon™, Franklin Lakes, New Jersey, US; 1:10 dilution) in FACS buffer containing PBS supplemented with 0.1% bovine serum albumin for 40–60 min at room temperature. Then, the cells were stained with the secondary antibody goat-anti mouse for 15–30 min at room temperature. Cells were washed with FACS buffer and sorted on a FACSAria cell sorter in the high-purity single-cell mode (Becton Dickinson) and then analyzed by a FACS Canto II flow cytometer (Becton Dickinson) and FCS express software (De Novo software, Ontario, Canada).

RNA-Sequencing Data Analyses

Sample preparation of primary PDAC cells was performed as described previously and sequenced using 100 bp SE with Illumina Platform [4]. The raw data were preprocessed for quality filtering and adapter trimming using FASTX Toolkit (version 0.7) and subsequently mapped to the Human genome (GRCh38) using STAR alignment tool (version 2.5.3a) [5,6]. We obtained ~90% of reads mapped to the Human Genome per sample. Gene counts in FPKM normalization were computed using CuffLinks algorithm and plots were generated with R version 3.5.0 [7].

Measurement of Serum HGF levels in PDAC Patients

The concentration of HGF was measured in serum from patients with metastatic PDAC, treated within a dose-finding phase I trial of gemcitabine given as prolonged fixed dose rate infusion [8]. In particular we used the serum samples (50 μL) collected from five patients who achieved stable disease and five patients who underwent progression at restaging of disease, which was performed by CT after eight courses of treatment. The patients were required to sign an informed consent and the Ethics Committee of St. Chiara University Hospital approved the study. For the analysis we used the ELISA Kit (Human HGF Quantikine ELISA Kit, R&D Systems, MN, USA), which was used for the analysis of the cell medium, following the manufacturer's instructions.

References

1. Gottschling, S.; Jensen, K.; Warth, A.; Herth, F.J.F.; Thomas, M.; Schnabel, P.A.; Herpel, E. Stage-specific embryonic antigen-4 is expressed in basaloid lung cancer and associated with poor prognosis. *Eur. Respir. J.* **2013**, *41*, 656–663.
2. Lou, Y.-W.; Wang, P.-Y.; Yeh, S.-C.; Chuang, P.-K.; Li, S.-T.; Wu, C.-Y.; Khoo, K.-H.; Hsiao, M.; Hsu, T.-L.; Wong, C.-H. Stage-specific embryonic antigen-4 as a potential therapeutic target in glioblastoma multiforme and other cancers. *Proc. Natl. Acad. Sci. USA* **2014**, *111*, 2482–2487.
3. Afrikanova, I.; Kayali, A.; Lopez, A.; Hayek, A. Is stage-specific embryonic antigen 4 a marker for human ductal stem/progenitor cells? *Biores. Open Access* **2012**, *1*, 184–191.
4. Sciarrillo, R.; Wojtuszkiewicz, A.; Kooi, I.E.; Gómez, V.E.; Boggi, U.; Ansen, G.; Kaspers, G.-J.; Cloos, J.; Giovannetti, E. Using rna-sequencing to detect novel splice variants related to drug resistance in in vitro cancer models. *J. Vis. Exp.* **2016**, doi: 10.3791/54714.
5. Gordon, A.; Hannon, G.J. Fastx-toolkit. http://hannonlab.cshl.edu/fastx_toolkit/index.html
6. Dobin, A.; Davis, C.A.; Zaleski, C.; Schlesinger, F.; Drenkow, J.; Chaisson, M.; Batut, P.; Jha, S.; Gingeras, T.R. Star: Ultrafast universal rna-seq aligner. *Bioinformatics* **2012**, *29*, 15–21.
7. Roberts, A.; Trapnell, C.; Donaghey, J.; Rinn, J.L.; Pachter, L. Improving rna-seq expression estimates by correcting for fragment bias. *Genome Biol.* **2011**, *12*, doi: 10.1186/gb-2011-12-3-r22.
8. Bengala, C.; Guarneri, V.; Giovannetti, E.; Lencioni, M.; Fontana, E.; Mey, V.; Fontana, A.; Boggi, U.; Del Chiaro, M.; Danesi, R.; et al. Prolonged fixed dose rate infusion of gemcitabine with autologous haemopoietic support in advanced pancreatic adenocarcinoma. *Br. J. Cancer* **2005**, *93*, 35–40.
